# Women at Altitude: Sex-Related Physiological Responses to Exercise in Hypoxia

**DOI:** 10.1007/s40279-023-01954-6

**Published:** 2023-10-30

**Authors:** Antoine Raberin, Johannes Burtscher, Tom Citherlet, Giorgio Manferdelli, Bastien Krumm, Nicolas Bourdillon, Juliana Antero, Letizia Rasica, Davide Malatesta, Franck Brocherie, Martin Burtscher, Grégoire P. Millet

**Affiliations:** 1 Institute of Sport Sciences, Faculty of Biology and Medicine, University of Lausanne, Lausanne, Switzerland; 2 Laboratory Sport, Expertise and Performance (EA 7370), French Institute of Sport, Paris, France; 3 Institut de Recherche Bio-Médicale Et d’Épidémiologie du Sport (EA 7329), French Institute of Sport, Paris, France; 4 Faculty of Kinesiology, University of Calgary, Calgary, Canada; 5 Department of Sport Science, University of Innsbruck, Innsbruck, Austria

## Abstract

Sex differences in physiological responses to various stressors, including exercise, have been well documented. However, the specific impact of these differences on exposure to hypoxia, both at rest and during exercise, has remained underexplored. Many studies on the physiological responses to hypoxia have either excluded women or included only a limited number without analyzing sex-related differences. To address this gap, this comprehensive review conducted an extensive literature search to examine changes in physiological functions related to oxygen transport and consumption in hypoxic conditions. The review encompasses various aspects, including ventilatory responses, cardiovascular adjustments, hematological alterations, muscle metabolism shifts, and autonomic function modifications. Furthermore, it delves into the influence of sex hormones, which evolve throughout life, encompassing considerations related to the menstrual cycle and menopause. Among these physiological functions, the ventilatory response to exercise emerges as one of the most sex-sensitive factors that may modify reactions to hypoxia. While no significant sex-based differences were observed in cardiac hemodynamic changes during hypoxia, there is evidence of greater vascular reactivity in women, particularly at rest or when combined with exercise. Consequently, a diffusive mechanism appears to be implicated in sex-related variations in responses to hypoxia. Despite well-established sex disparities in hematological parameters, both acute and chronic hematological responses to hypoxia do not seem to differ significantly between sexes. However, it is important to note that these responses are sensitive to fluctuations in sex hormones, and further investigation is needed to elucidate the impact of the menstrual cycle and menopause on physiological responses to hypoxia.

## Introduction

Systemic hypoxia refers to an environment with a reduced oxygen (O_2_) availability that arises from a decreased barometric pressure (hypobaric hypoxia) or a reduced ambient O_2_ concentration, which leads to lower inspired O_2_ fraction (F_I_O_2_) (normobaric hypoxia), resulting in an inspired partial pressure of oxygen lower than 150 mmHg [1]. As a consequence, steps involved in the O_2_ transport, known as the O_2_ cascade, are altered [2], and without appropriate responses, O_2_ supply may be compromised. Hence, to maintain an acceptable O_2_ supply, humans have developed many adaptations that are dependent on hypoxic stress. The duration, severity, type, and intermittent pattern of the exposure to hypoxia modulate the acute responses or long-term adaptations [3, 4].

Over 500 million humans live above 1500 m, which represent about 7% of the total world population [5]. Moreover, a growing number of individuals are exposed to altitude/hypoxia in different scenarios, such as mountain tourism (e.g., mountaineering, trekking), winter sports, air travel, altitude training camps, or hypoxic training (i.e., tents/rooms at sea level). Therefore, adaptations to hypoxia have been investigated and described by researchers during the past decades [3, 6, 7]. However, despite the huge amount of literature regarding the specific responses to hypoxia, most studies were conducted in men [8]. Only a few studies have investigated putative sex-related responses to altitude and these were mainly limited to ventilatory [9, 10] or endocrine [11] functions. Overall, the underlying mechanisms of sex-related specificities remain scarcely investigated. To our knowledge, there is no comprehensive review covering the main sex-related physiological responses to hypoxia. Therefore, this review first draws upon an extensive literature search on five sex-related responses implied in oxygen cascade changes in hypoxia (i.e., respiratory, hemodynamic, haematological, muscle metabolism, and autonomic responses). We also discuss the impact of hypoxia on exercise at altitude and the influence of sex hormone changes with menstrual cycle and menopause. While slight differences in the physiological responses to normobaric versus hypobaric hypoxia have been reported [12], both types of hypoxic exposure are considered in this review due to data scarcity. The impact of the type of exposure is identified and discussed whenever a difference is shown or suspected.

## Physiological Mechanisms to Sex-Related Differences in Hypoxia

### Respiratory Responses

The pulmonary system plays a central role in both acute and chronic responses to hypoxia as the O_2_ provider of the organism. An increase in ventilation, known as the hypoxic ventilatory response (HVR), occurs rapidly during hypoxic exposure to maintain a functional alveolar to arterial O_2_ pressure gradient, and consequently reduces the alteration in O_2_ pulmonary diffusion. This HVR response is driven by chemoreceptors and is highly variable among individuals. The impact of sex on HVR is not fully understood, with contradictory studies reporting either higher [13], lower [14], or similar [15] HVR between men and women. A recent study on a large cohort reported a lower HVR in women even after correction for body surface area [16]. These inconsistent findings regarding HVR could be explained by the impact of sexual hormones on ventilation and hence the menstrual cycle since sex hormone receptors are located in the carotid body, which contains the primary chemoreceptors monitoring blood oxygen levels [17, 18] (peripheral chemoreceptors, see Sect. 3.1). Central chemoreceptors, located throughout the lower brainstem, modulate respiration based on changes in CO_2_ and consequently in pH. Low chemosensitivity could greatly contribute to sex-related specific responses to hypoxia. This is suspected to be involved in acute mountain sickness (AMS) [19] and relative hypoventilation during exercise [20]. Moreover, it was recently demonstrated that women had a lower hypercapnic ventilatory response (i.e., lower central chemosensitivity) than men at rest [21] and during aerobic exercise [22], which may contribute to relative hypoventilation.

Relative hypoventilation (with shunt, ventilation-to-perfusion mismatch, diffusion limitation, and insufficient red blood cell transit time) is one of the underlying mechanisms of exercise-induced hypoxemia (EIH) [20]. Hypoxemia refers to a decrease in O_2_ blood content and is a cause of the reduced O_2_ availability. The EIH phenomenon observed at sea level is suspected to be more common in women [23] than in men [24] during a maximal cycling exercise (67% versus 52%; respectively), despite the lack of temperature-corrected arterial blood gases. However, greater pulmonary gas exchange impairment in women has not always been reported [25, 26]. Moreover, while EIH is exclusively observed in highly trained male endurance athletes [24], it has been reported in both trained and untrained women [23, 27, 28]. Although the consequences of EIH during altitude exposure are not fully understood, higher oxidative stress [29], lower pulmonary vascular resistance [30], and lower O_2_ arterial saturation (S_a_O_2_) [31] have been reported in individuals exhibiting EIH when exposed to altitude. It is known that individuals with EIH have a larger drop in maximal O_2_ uptake (*V*O_2max_) during exercise in acute hypoxia [31, 32]. Indeed, a relationship between EIH severity (i.e., lowest S_a_O_2_ in normoxia) and *V̇*O_2max_ decline in hypoxia was reported even at a low altitude (1000 m) [32]. Knowing that even untrained women are susceptible to developing EIH and that EIH may be more prevalent in female athletes, one may speculate that, on average, women may exhibit a greater hypoxia-related decline in *V̇*O_2max_ than men. Moreover, women seemed to be more hypoxemic than men when exposed to the same hypoxic intensity and duration because of poor ventilatory response, right shift in the oxyhaemoglobin dissociation curve, and a lower circulating capacity and reservoir [i.e., lower body surface area and lower normalized blood volume and haemoglobin mass (Hb_mass_)] [33–35].

Apart from reduced chemosensitivity, hypoventilation may also be due to mechanical constraints such as expiratory flow limitation (EFL) [27]. EFL occurs when ventilatory demands reach ventilatory capacity and refers to the inability to generate higher expired flow despite increased expiratory effort [36]. Women are known to have proportionally smaller lung volumes and airways than men [37], which lowers their ventilatory capacity. Lung size is related to standing height, and since men are taller than women, population-based studies indicate that women have smaller absolute lung volume even when matched for height with men [38]. As a consequence, absolute gas exchange surface and pulmonary capillary volume are reduced as well as lung diffusion capacity (i.e., the ability of the pulmonary system to allow effective gas transfer between alveoli and pulmonary capillaries) [39]. A reduced diffusion capacity in women, which is known to change at rest [40] and during exercise [41] across the menstrual cycle, may affect pulmonary gas exchange under hypoxic conditions. However, the difference in lung diffusion capacity disappears when corrected for lung size and cardiac output [26, 39]. Therefore, women did not display higher diffusion limitation in normoxia and hypoxia (F_I_O_2_ = 0.125) [26]. This highlights the importance of absolute lung volume in determining pulmonary limitation to exercise, particularly when comparing women and men. Moreover, since hypoxic pulmonary vasoconstriction is one of the first adaptations to a hypoxic environment [42], its influence on sex-related differences in diffusion capacity remains to be characterized, especially given the attenuated pulmonary artery vasoreactivity in hypoxia when circulating oestrogen level is elevated [43]. Women also have a smaller large-conduit airway than men, which impacts flow resistance [44]. Airflow passes from laminar to turbulent flow when air velocity (determined by ventilation) increases. In this context, a small airway cross-sectional area favors turbulent flow, which increases the resistive work of breathing (*W*_b_) [45]. Hence, women have an increased W_b_ compared with men [46], and one can hypothesize that the hypobaric-related decrease in air density at altitude may induce greater benefits in women (only during hypobaric exposure). During exercise, when women breathed a Heliox gas mixture (21% O_2_ balance with Heliox), which is less dense than air, their *W*_b_ was similar to that of men breathing room air [46]. However, the reduction of air density is likely large enough to obtain such a benefit only at very high altitude, since individuals should be exposed to an altitude over ~ 5500 m to reach the Heliox gas mixture density. To our knowledge, although several differences in physiological responses to normobaric versus hypobaric hypoxia have been reported [12], there are no data comparing men and women exposed to these two hypoxic conditions.

Due to their above-described higher *W*_b_, women exhibit greater respiratory muscle *V̇*O_2_ and are thought to be more susceptible to exercise-induced diaphragm fatigue. A recent study showed that diaphragm fatigue was not impacted by sex in normoxia, while in hypoxia healthy women were more susceptible to this fatigue when compared with men [47]. However, during whole body exercise, diaphragm fatigue was not different between men and women in normoxia and hypoxia, but diaphragm recovery in hypoxia was impaired in women [48]. An efficient strategy to limit this exacerbated diaphragm fatigue in hypoxia consists of respiratory muscle training (RMT). A greater improvement of endurance performance in hypoxia compared with normoxia was reported after 4 weeks (20 sessions) of RMT [49]. Of importance, this performance enhancement was greater in women than in men. Since a substantial percent increase in *V̇*O_2max_ following altitude training goes to fuel the respiratory muscles [50], one may speculate that the benefits of RMT prior to or during altitude exposure would be larger and may induce a greater post-altitude *V̇*O_2max_ increase in women than in men.

Overall, the female pulmonary system seems more detrimentally impacted by altitude due to a higher propensity to exhibit EIH and exacerbated diaphragm fatigue in hypoxia. The difficulty in drawing definite conclusions regarding pulmonary function in hypoxia is that lung size is impacted by sex; hence normalization could abolish potential sex differences. Moreover, many of these assumptions are drawn from studies including only few women. Therefore, further studies with specific attention toward sex-related pulmonary system responses to hypoxia are required.

### Cardiac and Hemodynamic Responses

Growing evidence demonstrates sex differences in cardiac hemodynamic and vascular regulation, both at rest and during exercise, which have direct consequences on the responses to hypoxia.

Across all ages, at rest, women present similar [51] or higher heart rate (HR) [52] and lower absolute stroke volume (SV) and cardiac output (*Q̇*_C_), but higher peripheral resistance, compared with men [51, 53]. However, when body surface area is taken into account, no sex differences are reported for SV and peripheral resistance, though contrasting results still persist regarding *Q̇*_C_ indexed for body surface area [51, 53]. When normalized for body surface area, differences in peripheral vascular tone disappear; this mechanism explains why the higher absolute total peripheral resistance in females remains debated. In women, contrary to men, the greater peripheral vascular tone at rest seems to be unrelated to sympathetic activity [51]. There are several candidates for sex differences in cardiovascular function: greater levels of circulating endogenous nonadrenergic vasoconstrictors [51], lower release of vasoconstrictors (primarily norepinephrine and/or neuropeptide Y) per burst of sympathetic traffic [51], and/or specific effects of female sex hormones (i.e., estrogens) that may offset sympathetically mediated vasoconstriction [54, 55].

The differences in resting blood pressure between women and men are more complex since menopause reverses the relationship between blood pressure and age [56]. From youth to middle age, men consistently have significantly higher systolic and diastolic pressures compared with women [51, 52]. After the age of 55 years, due to a greater increase in systolic blood pressure and similar decrease in diastolic blood pressure as compared with men, healthy women present a larger increase in pulse pressure (i.e., the difference between systolic and diastolic blood pressures, which is an index of arterial stiffness) [52, 56].

During exercise, HR, *Q̇*_C_, and blood pressure increase due to sympathetic stimulation, catecholamines releases, and vasodilation occurs in contracting muscles. Even when matched for body size, females demonstrated divergent cardiovascular responses to dynamic exercise compared with men [57], as demonstrated by a more rapid increase in blood pressure and HR, and a smaller exercise-induced increase in ejection fraction [58, 59]. Overall, it has been suggested that females have a greater reliance on HR to meet the metabolic demands of exercise, whereas men rely on preload and enhanced use of the Frank–Starling mechanism to increase *Q̇*_C_ [59]. Moreover, premenopausal women (i.e., start of menarche to start of the menopause) demonstrate a greater vasodilatory capacity during incremental leg exercise compared with men [60] and lower sympathetic vasoconstriction responsiveness to pharmacological and nonpharmacological stimuli [61, 62], suggesting that β-adrenergic receptors are either more sensitive or upregulated in premenopausal women versus age-matched men.

Hypoxia also represents an important stressor for the cardiovascular system. The cardiac and blood flow control response is a dynamic process that progresses over hours, days, and weeks at altitude, and it appears to be largely driven by stimulation of the sympathetic nervous system. During acute exposure to altitude (> 700 m [63]), sympathetic stimulation aims to increase HR, SV, and systemic blood flow (i.e., *Q̇*_C_) proportionally to the degree of hypoxia [64, 65]. With prolonged exposure, gradual systemic adaptations (i.e., decreases in SV and *Q̇*_C_ despite an increased HR) occur to attempt to restore cardiovascular function toward normoxic levels [66, 67]. Substantial evidence suggests that differences in resting cardiac hemodynamic responses and vascular regulation during both acute and prolonged exposure to altitude might be influenced by sex [63, 64, 68, 69]. Specifically, while resting HR and blood pressure increase similarly between women and men acutely exposed to hypoxia [69, 70], there are contrasting results for sex differences in resting blood flow and vascular conductance between femoral and forearm vasculature [69, 70]. An emerging body of literature suggests that premenopausal women exhibit a different peripheral blood flow response (i.e., attenuated vasoconstriction or enhanced vasodilation) to physiological stressors, including acute exposure to hypoxia, when compared with men [68–72]. Indeed, substantial sex differences exist in regional blood flow and compensatory vasodilatory response to hypoxic exercise. Casey and colleagues were the first to report a greater compensatory vasodilatory response to submaximal handgrip exercise (10% and 20% of maximal voluntary contraction) in premenopausal, but not in older, females compared with males [69]. While female sex hormones likely influence these responses, it was suggested that premenopausal women present lower sympathetic vasoconstrictor activity during hypoxic exercise compared with men, and therefore a greater compensatory vasodilation [69]. Moreover, nitric oxide (NO) represents a major vasodilatory signaling molecule released in response to acute hypoxia [73], with the exception of the pulmonary vasculature where hypoxia elicits vascular constriction [74], and sex-specific differences in NO release and NO-dependent vasodilation may exist. Early animal models suggested greater NO-mediated sympatholysis and sympathetic vasoconstriction at rest in females compared with male rats [75]. More recently, in humans, females showed similar resting cutaneous microvascular NO-dependent vasodilation to men. However, greater microvascular NO-dependent vasodilation was found in eumenorrheic women compared with both women using oral contraceptives and men [76]. Nevertheless, sex differences in the contribution of NO to hypoxia- or exercise-induced vasodilation remain to be further elucidated.

Exercise at altitude (acute exposure) increases HR but not SV, leading to an increased *Q̇*_C_ compared with the same exercise performed at sea level [77, 78]. However, the literature is still scarce about HR, SV, and *Q̇*_C_ responses to hypoxic exercise in females versus males.

In conclusion, substantial sex differences exist between premenopausal women and men in vascular control, rather than in the cardiac hemodynamic responses, during acute exposure to hypoxia at rest or in combination with exercise. These responses seem to be primarily mediated by lower activation of exercise- and hypoxia-induced β-adrenergic receptors or blunted sympathetic vasoconstriction observed in premenopausal females compared with males.

### Hematological Responses

Plasma volume (PV) contraction is one of the first hematological responses to a hypoxic environment [79]; hence, significantly higher haemoconcentration is observed in altitude for lower basal values [80]. This hemoconcentration, which arises from a reduction in total circulating plasma protein mass (TCPP) rather than a fluid loss [81], is similar between men and women [82]. It has a direct impact on hemoglobin concentration (Hb) and hematocrit (Hct) levels. Despite lower average values in females [83], mainly caused by the effect of sex hormones on erythropoiesis [84], acute hematological changes seem to be sex independent [85]. However, women apparently reach maximal Hb and Hct values more rapidly (approximately 5 days, compared with 7 days in men) during exposure to the same altitude [80].

Consecutive to the above-described initial hematological adaptations, the increase in erythropoietic activity is proportional to the severity of the hypoxic stimulation [86] and therefore determined by the hypoxic dose (e.g., the altitude and duration of exposure) [87]. Erythropoietin (EPO) is the key hormone in the erythropoietic process [88] due to the upregulation of the hypoxia-inducible factor (HIF) [89]. Serum EPO tends to increase drastically during the first 3 days at altitude before progressively declining, with a return to basal values after 1–3 weeks [90]. Despite a large interindividual variability [91], the EPO time course at altitude seems to be very similar between women and men [92–94].

Several days are required before an increase in reticulocytes is detected, frequently followed by an expansion of the total Hb_mass_ [95]. A greater within-subject variability in reticulocyte level was observed in female athletes, potentially explained by the influence of the menstrual cycle [96]. Although the menstrual cycle is known to impact multiple blood variables such as Hb and reticulocyte parameters [97], study of the menstrual cycle in relation to hematological adaptations at altitude remains poorly documented. An Hb_mass_ expansion of ∼1.0 to 1.1% per 100 h of altitude exposure is commonly expected in male athletes [86, 98]. In line with the latter assertion, 12 nights of normobaric hypoxia were insufficient to detect a significant Hb_mass_ increase in elite female athletes [99], with the hypoxic dose probably being too low. Following a sigmoidal pattern, a stabilization of Hb_mass_ is usually observed after 3 weeks of exposure [100]. As the decrease in PV is only partially compensated for by the progressive increase of Hb_mass_, a decrease in blood volume is usually observed during the first weeks of exposure [101]. No clear consensus is apparent regarding the impact of sex on Hb_mass_ increase. While some results did not report differences between sexes [92, 93], a recent study showed a smaller increase in female athletes [102], although the latter results are probably partially explained by a low initial level of s-ferritin in the female cohort (< 30 μg L^−1^). Furthermore, the influence of the initial Hb_mass_ level on the potential gains does not seem to be fully understood, with contradictory results [103]. However, since the response to altitude training between the sexes has not been systematically studied, in addition to a smaller number of women commonly included in the studies [102], the putative sex-related differences remain unclear and need to be further investigated.

Overall, most of the results do not show any large impact of sex on erythropoietic adaptation [91, 93, 104]. Nevertheless, a difference was observed among altitude residents, reporting a lower Hb_mass_ level in women than in men [105]. At that time, a better ventilatory response leading to better arterial oxygenation and a consequently lower erythrocyte requirement in women was suggested by the authors. These mechanisms are not supported by the recent literature (see Sect. 2.1). Moreover, a later study did not confirm these mechanisms and reported a similar increase in Hb_mass_ between pre- and postmenopausal women living at 2600 m [106].

While the classification of altitude responders and nonresponders remains debated [91, 102], it is well known that iron stores, with increasing demand at altitude [107], are an essential component of hematological adaptations [90, 104]. With a higher prevalence of iron deficiency in female athletes (15–35%) [108], this population could be more at risk for reduced hematological adaptations and this needs to be further investigated. Adequate pre-altitude iron stores are needed for hematological adaptations during altitude exposure [109, 110]. Therefore, a critical point relates to iron status, with an adequate baseline s-ferritin (> 30 ng/mL for women and > 40 ng/mL for men) being assumed to support the general evidence of progressive enhancement of Hb_mass_ with altitude/hypoxia exposure [95, 109–113].

However, substantial Hb_mass_ increases can be achieved even with low pre-altitude s-ferritin, provided adequate iron supplementation is implemented at altitude [109, 114]. Koivisto-Mørk et al. [115] recently showed that pre-altitude s-ferritin or iron supplementation (which prevent a decrease in s-ferritin at altitude) were not the limiting factors for an altitude-induced increase in Hb_mass_ (+ 3.7%) in world-class endurance athletes with clinically normal iron status. Whether iron treatment is appropriate or not for female athletes with nonanemic iron deficiency (s-ferritin < 30 μg L^−1^, with all other hematological variables being normal) still remains unresolved [116].

### Muscle Metabolism

Beyond the sex differences in ventilatory and hemodynamic responses to hypoxia, distinct peripheral responses due to different physiological muscle properties have been identified between females and males.

For instance, studies reported that females have a higher portion of type I fibers in the vastus lateralis [117, 118], although males showed a larger fiber cross-sectional area [119].

In the literature, mixed results have been reported when sex differences in muscle capillarization are investigated [120, 121], but greater microvascular reactivity has been found in males compared with females [122]. Moreover, equivocal data are presented on skeletal muscle oxidative capacity [123, 124], even if the literature supports no differences between males and females when the populations are matched for fitness level and lean body mass [125, 126]. The magnitude of increase in muscle deoxygenation measured by near-infrared spectroscopy placed on vastus lateralis during repeated sprint exercise appeared to be significantly lower for females than males [127], supporting the current knowledge of a more efficient oxidative metabolism in females than males. However, this should be interpreted with caution because of the higher body fat mass reported in women that can confound the near-infrared spectroscopy signal [128]. Indeed, consistent results are reported on sex influences in skeletal muscle metabolism, with females oxidizing more fat and less carbohydrate and amino acids during endurance exercise compared with males [129], probably as a consequence of the higher portion of slow oxidative fibers and lower glycolytic enzyme activity [130], in association with higher levels of 17-β-estradiol, which mediates lipid oxidation [131].

The possible implications of the above-reported sex differences in oxygen utilization in hypoxic conditions have been only marginally explored. The shift in substrate preference in hypoxia (i.e., a decreased reliance on free fatty acids and an increase in glucose dependence) is well known [132, 133]. Sandoval and Matt investigated sex differences in metabolic substrate utilization in hypoxia, with females shifting toward greater fat use and males shifting toward greater carbohydrate use during exercise [134]. However, the sex differences in the metabolic shift in hypoxia remain largely unexplored, along with the possible impact of these characteristics on hypoxic training interventions.

**Fig. 1:**
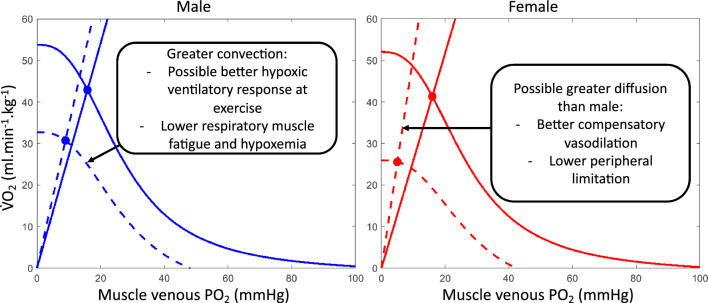
Schematic representation of altitude-related changes in convective (calculated from *V̇*O_2_ = cardiac output × difference in arterio-venous O_2_ content, sigmoid line, [236]) and diffusive (calculated from *V̇*O_2_ = diffusion coefficient × mixed venous O_2_ pressure, straight line, [236]) components of *V̇*O_2max_ in one male and female individual matched for sea-level *V̇*O_2max_. The full line is in normoxia, the dotted line is in hypoxia (5260 m). The individual datasets are from [237]. Cardiac output was computed with pulse wave contouring analysis. The convective component was reduced to a larger extent in female individual due to a higher altitude-induced hypoxemia and lower hemoglobin concentration, while no clear differences in cardiac hemodynamic responses were noted. Due to a higher compensatory vasodilation and lower sympathetic vasoconstrictor activity, the diffusive component of *V̇*O_2max_ was improved in female individuals in hypoxia. Therefore, female individuals seem more centrally but less peripherally limited than men when exercising in hypoxia. *V̇O*_*2*_ oxygen uptake, *PO*_*2*_ oxygen pressure

The pioneering work of Shephard and colleagues [135] demonstrated that the differences between males and females in maximal oxygen consumption in hypoxia are significantly decreased when the volume of active muscles is reduced during specific modalities of exercise (two-legged versus one-legged exercise). Reducing the active muscles during exercise makes peripheral limitations predominant in determining endurance performance [136], and when this condition is achieved by studying upper limb exercises, females seemed to have no impairment in hypoxic compared with normoxic exercise conditions [135]. This work suggests that the exercise capacity of the arms per unit volume of muscle in hypoxia is greater in females compared with males, but more studies are needed to confirm this and investigate the possible impact of peripheral sex differences in oxygen utilization. The above-described mechanisms may alter O_2_ transport and consumption. Convective and diffusive components of *V̇*O_2max_ in normoxia and hypoxia are displayed with representative data in Fig. 1. This method should help to highlight the cause of *V̇*O_2max_ limitations in female and male individuals in hypoxia.

### Autonomic Responses

One critical response to hypoxia is the modulation of the autonomous nervous system activity, with an increased sympathetic activation concomitant with a parasympathetic withdrawal [137, 138]. Several studies reported sex differences in autonomic function and its hypoxia-induced modulation.

In normoxia, the sex differences in various parameters associated with autonomic function are divergent. In premenopausal women, cerebral autoregulation has been reported to be similar to [139, 140], more efficient than [141, 142], or less efficient than age-matched men [143]. Women show greater vagal modulation in heart rate variability (HRV) in normoxia [144]. However, whether the cerebral autoregulation, which is partially dependent on vagal modulation, is related to HRV in hypoxia remains unclear. Comparably, there was no clear difference in baroreflex function in premenopausal women and age-matched men, yet baroreflex sensitivity seemed blunted in older women (> 40 years old) [145]. Moreover, women were shown to have a smaller increase in sympathetic indices and cerebrovascular resistance index, and greater parasympathetic withdrawal and vasodilation during the tilt test than men [146–148].

Sex-based vascular differences have already been described (see Sect. 2.2). The response may differ between vascular beds, with sex-related differences being reported in the forearm circulation [71, 149] but absent in the legs [70]. Systemic vascular responses may not differ, whereas responsiveness within the mesenteric circulation is sex specific [150] and seemed neurally mediated [72]. Hypoxia differentially modulates vascular responsiveness to sympathetic activation in men and women [151], which may partly explain the differences observed in AMS susceptibility [9].

In hypoxia, muscle sympathetic nerve activity (MSNA) responses were augmented in premenopausal women compared with men, indicating an augmented sympathetic response to both central chemoreflex and combined peripheral and central chemoreflex activation [21]. These findings may be associated with the reduced ventilatory response, as described in Sect. 2.1. However, the causal link between the two phenomena remains unexplored [21]. In addition, sex differences in the respiratory–sympathetic coupling likely depend on intrinsic properties of the respiratory–sympathetic network [152]. This may be one underlying mechanism of the higher diaphragm fatigue described above in women [47] (Sect. 2.1). The sympathetic activity has a rhythmic component associated with the respiratory cycle [153], in which the sympathetic discharge occurs at the end of inspiration and the beginning of postinspiration [154, 155]. This respiratory–sympathetic coupling is mainly due to synaptic interactions between respiratory and presympathetic neurons at the rostral ventral medulla [156, 157]. Changes in the respiratory–sympathetic coupling may lead to increased sympathetic activity and may partially account for some of the sex differences listed above.

## Impact of Hormonal Changes

### Menstrual Cycle Variation

**Fig. 2:**
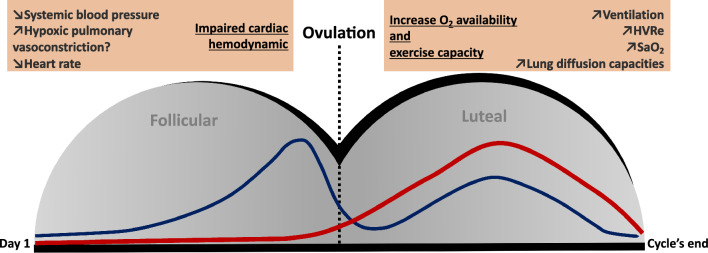
Schematic representation of estrogen (blue curve) and progesterone (red curve) expected in eumenorrheic women and their potential influence on altitude-related physiological responses. The transition from follicular to luteal phase is determined by ovulation. The gray half circles represent each phase and the black line illustrates the schematic representation of the hormonal environment (related to the sum of estrogens and progesterone). *HVRe* hypoxic ventilatory response at exercise, *SaO*_*2*_ oxygen arterial saturation

The different endocrine environments between females and males promoted by estrogen, progesterone, testosterone, and their precursors influence human physiology and sex differences [158]. In eumenorrheic women, these hormones fluctuate along the menstrual cycle phases (Fig. 2). The ovarian cycle starts with the first day of bleeding, when the sex hormone levels are at their lowest. Estrogen levels increase during the follicular phase and peak just before the surge of luteinizing hormone, stimulating ovulation that marks the transition to the luteal phase. During this phase, both estrogen and progesterone will increase and decrease shortly before the next bleeding in the absence of fertilization [159]. In women taking combined contraceptive pills (mono-, bi-, tri-, or quadriphasic oral contraceptives) the exogenous hormones are fairly constant and reduce the endogenous levels of sex hormones, followed by a phase without exogenous hormones during pill withdrawal. These sex hormone fluctuations impact numerous physiological functions that may influence training [160, 161] and performance [162, 163], such as recovery [164], wellness [165], and in particular, considering the altitude environment, respiratory function [166].

In normoxic conditions, an increase in ventilation, basal temperature, and resting HR has been well established during the luteal phase when progesterone peaks [167, 168]. With the use of combined oral contraception, an increase in ventilation, breathing frequency, and oxygen ventilatory equivalent occurs during the hormonal phase in comparison with the phase of pill withdrawal or nonhormonal phase [168].

Sex hormones may play a key role in the responses to hypoxic exercise depending on the ovarian cycle phase and menopausal status [9]. Studies have reported variations in ventilatory measures across the menstrual cycle [169–171]. Specifically, progesterone, acting both on peripheral and central chemoreceptors, has been suggested to be a potent respiratory stimulant [172]. The suggested mechanism is a reduced threshold of the medullary respiratory center, increasing its excitability [168]. There is ample evidence relating progesterone to the increase in resting minute ventilation during the mid-luteal phase [173, 174], favoring better oxygenation at high altitudes [175].

Some studies have hypothesized an increase in ventilatory levels, and ultimately in performance, in hypoxic conditions during the luteal phase. However, there is not a clear-cut answer to such a hypothesis. There are sparse studies relating the menstrual cycle to altitude performance, and the methods employed to define each phase (with or without hormonal measurements), the altitude level, the training status of the subjects, and the measured parameters differ widely, reducing comparability. The studied female population also widely ranges, from sedentary mice to trained women, from acclimated to high altitude to lowlanders tested at rest or in exercising conditions [9, 10, 15, 174, 176–179].

A study including a large cohort, with 1060 healthy women showed that HVR at exercise (HVRe), which is a determinant factor of tolerance to high altitude, depends on the ovarian cycle phase [9]. As hypothesized, HVRe was maximal in the early luteal/mid-luteal phase, when estrogen and progesterone were high in comparison to the early follicular phase, suggesting optimal oxygenation and ventilatory adjustments under such hormonal milieu. Oral contraception or hormonal treatment had no effect on ventilatory responses to hypoxia [9]. HVR was also shown to be higher during pregnancy [180, 181], when progesterone levels increase; while ovariectomy, which decreases natural hormones production, decreased HVR [182]. Another study, based on nine trained women performing a maximal exercise test, showed that the ratio of minute ventilation and oxygen uptake did not differ across the menstrual cycle phases at sea level, but was greater in the luteal phase than in the follicular phase under hypobaric hypoxia conditions (equivalent to 3000 m), both at rest and during peak exercise [177]. In addition, the partial pressure of end-tidal carbon dioxide during exercise was lower in the luteal phase, indicating that a hyperventilatory response occurred during peak exercise [177].

This HVR increase during the luteal phase, with stimulated breathing associated with sex hormones, seems a robust finding [14, 183–185]. Yet studies based on ten women with hormonal measurements to precisely discriminate the cycle phases, showed no differences in the ventilatory response to hypoxic exercise across the cycle [15, 174]. Also, when comparing women to men, no clear differences in HVR were found [184, 186–192].

The potential ventilatory impact on performance assessed through *V̇*O_2max_ in healthy women is yet to be demonstrated, with most studies failing to show significant differences among the hormonal phases [174, 176, 177]. This could be related to compensatory mechanisms allowing reaching of similar performance levels during the cycle through different parameter adjustments occurring in each phase, as has been shown to occur for cognitive tasks across the menstrual cycle [193]. Robust research, properly classifying the hormonal phases is needed, especially in hypoxic conditions.

Other physiological parameters have been shown to be affected by the menstrual cycle in hypoxic conditions, such an increase of oxygen saturation (SaO_2_) in the mid-luteal phase during exercise in hypoxic conditions [9] or with acute altitude exposure, but not large enough to affect submaximal exercise performance [174]. Hemodynamic pulmonary responses seem also to be dependent on the hormonal environment. Physiological increases in circulating estrogen levels in rats attenuated pulmonary artery vasoconstriction under both normoxic and hypoxic conditions, suggesting an effect of the menstrual cycle on the pulmonary artery vasoreactivity [43, 194]. Such findings have not been investigated in women. In agreement, it has been shown that lung diffusion capacities at exercise are lower during the early follicular phase (when estrogen levels are low) because of reduced pulmonary blood volume [41].

An investigation of the sympathoadrenal responses during acute high-altitude exposure in healthy women showed no differences in catecholamine levels during cycle phases but found higher blood pressure and HR during the luteal phase [195]. Regarding hypoxic cardiac response at exercise, no difference was found between cycle phases [9].

Previous studies have highlighted the need for additional robust studies to identify minor to moderate impacts on complex outcomes, such as performance, in association with highly variant parameters of the menstrual cycle [163, 196]. More research [197], relying on precise hormonal measurements (especially considering the potential alterations in the hormonal [198] and clinical profiles [199, 200] of the menstrual cycle at high altitudes), and on larger cohorts followed longitudinally, is needed to determine the coupled influence of menstrual cycle and altitude on performance.

Finally, an important clinical consideration is that menstruating people, particularly those with heavy menstrual bleeding, are at an elevated risk of iron deficiency, which can impair performance [201] especially in high-altitude environments [116]. Hence, screening for iron deficiency is widely recommended [202], notably before high training loads in hypoxic conditions for menstruating athletes.

For all these reasons, monitoring the menstrual cycle with a validated methodology [159] prior to competitions or altitude exposure may be valuable for optimizing hypoxia-induced benefits/performance. However, caution is needed. To date, there is no evidence on the effectiveness of such time-consuming protocols in women exposed to hypoxia/altitude.

### Pregnancy

Pregnancy is a unique physiological state characterized by significant changes in various systems to accommodate the growing fetus. It is estimated that almost half of pregnant women expose themself to altitude at some points during their pregnancy [203]. The risks associated with traveling to high altitudes during pregnancy have been reported as low [204]. Moreover, with adequate acclimatization, placental reserves can support exercise—even at vigorous intensity—at moderate altitude [205]. However, important alterations in fetal development and growth can occur in pregnant highlander women [206]. This section will nevertheless not discuss the effects of altitude on the fetus (which is beyond the scope of this review) and is limited to pregnant women exposed to hypoxia.

During pregnancy, the outward expansion of the ribcage to accommodate the growing uterus induces a decrease in functional residual capacity [45]. Adaptations related to female sex hormones, such as an increase in ventilation inducing hypocapnia [207] and an increase in HVR [180, 181], may also influence tolerance to hypoxia, as during the menstrual cycle (see Sect. 3.1). During hypoxic exposure, pregnancy accentuates the well-known acclimatization mechanisms, triggering additional hyperventilation, increases in cardiac output, and placental blood flow to ensure fetal oxygen supply. Intermediate acclimatization, marked by changes in 2,3-diphosphoglycerate and hemoglobin levels, typically takes effect over the course of days to weeks. The fetus also develops several adaptation mechanisms to face brief periods of hypoxia, as described elsewhere [205]. These acclimatization mechanisms maintain sufficient oxygenation in healthy pregnancies during acute exposure up to 4000 m [205]. Finally, pregnancy does not appear to affect the susceptibility to high-altitude illness [208].

### Aging and Menopause

Aging leads to different physiological changes. Menopause, in particular, marks the end of the reproductive life of women and is a life phase in which osteoporosis [209] and cardiovascular diseases [210] become more prevalent.

While the median age of menopause among different populations worldwide is ~ 50 years [211], several studies have reported a younger menopausal age at altitude [212–215]. It has been suggested that this could be due to increased gonadotropin stimulation, which increases the number of recruited follicles [216].

The increase in ventilation at altitude is a key adaptive mechanism. Richalet et al. [19], in their prospective cohort study, found that HVR to moderate exercise in hypoxia was the main physiological predictive parameter of AMS. The effect of age on HVR is debated: some studies reported an increase [217, 218], similar [219–224], or decrease in HVR [225–228] with aging. Overall, age does not seem to have a clear effect on HVR.

Even if age and sex seem to have no clear effect on HVR, one may expect that the change in sex hormones following menopause impacts HVR. Cistulli et al. [229] also reported increased HVR with estradiol treatment in postmenopausal women. However, most studies do not support this hypothesis: Pokorski and Marczak [221] found that HVR was not different between premenopausal and older healthy women, while Richalet and Lhuissier reported an increased HVR with aging in men but not in untrained postmenopausal women [230], and that HVR at exercise was similar in premenopausal versus postmenopausal women [9]. Overall, there is no evidence that menopause influences HVR.

Pulmonary function also declines with aging, which can impact the response to hypoxia. Indeed, loss of elastic recoil implies reduced ventilatory capacity and increased EFL in older men and women, but no sex-based comparison was performed [231]. Because of these mechanical constraints, EIH appears more frequently and at lower intensity in older individuals [232]. Although sex-related differences in EFL and EIH and their impact on performance in hypoxia were previously described (see Sect. 2.1), the putative cross effect of aging and sex on these pulmonary limitations to performance at altitude remains to be characterized. Aging is also known to induce a pulmonary vascular remodeling that led to slight progressive increase in resting pulmonary vascular resistance and hypertension [233]. These increases are more pronounced during exercise. While estrogen seems beneficial for pulmonary reactivity [43], women are more susceptible to pulmonary hypertension, a phenomenon known as the ‘estrogen paradox’ [234]. One may hypothesize that this loss of estrogen may alter pulmonary vasculature, making HPV more severe in older women than age-matched men.

Although premenopausal women have greater compensatory vasodilation in hypoxia compared with age-matched men, this sex difference disappears after menopause [69]. A reduction in estrogen could be suspected to be at the onset of this change since estrogen is known to modulate vascular tone in animal models during dynamic exercise [235] or hypoxia exposure [43]. However, greater compensatory vasodilation in hypoxia has been reported in women during their early follicular phase, which minimized the acute role of estrogen in this mechanism [69]. Hence the mechanism at the onset of the blunt vasodilatory response to hypoxia after menopause deserves further investigation.

In conclusion, (1) menopause occurs sooner in altitude; (2) age, sex, and menopause seem to have no effects on HVR; and (3) the age-related change in pulmonary and autonomic function could increase or reduce sex differences and may need more investigation.

## Conclusions

**Fig. 3:**
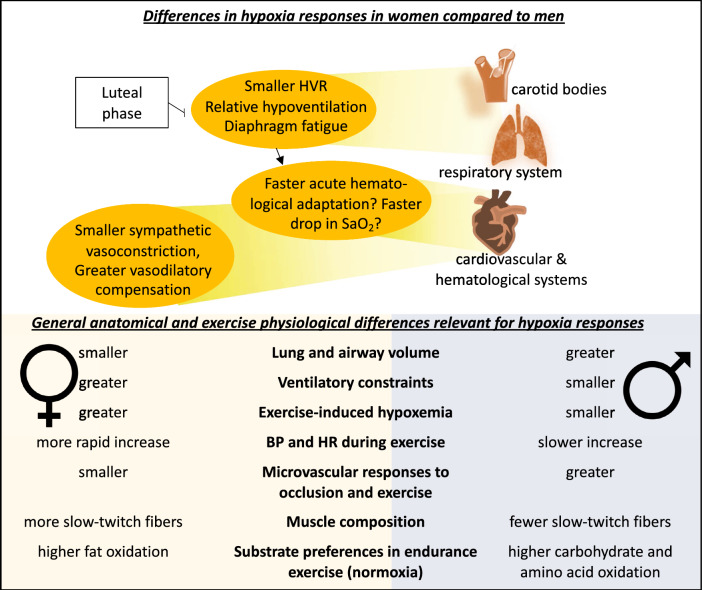
Mechanisms of sex-related differences in response to hypoxia. *HVR* hypoxic ventilatory response, *SaO*_*2*_ oxygen saturation, *BP* blood pressure, *HR* heart rate. References: Lung and airway volume [37, 38, 44], Ventilatory constraints [22, 23, 33], Exercise-induced hypoxemia [27, 28, 33], BP and HR during exercise [57–59], Microvascular responses to occlusion and exercise [122, 127], Muscle composition [130], Substrate preferences in endurance exercise [129]

In this review, we have emphasized the sex differences in responses to hypoxia (Fig. 3). The pulmonary system seems to be one of the functions most impacted by sex differences and exposure in hypoxia, with women becoming more hypoxemic and having a greater work of breathing than men. Cardiac hemodynamics are not impacted by sex in hypoxia, but vascular reactivity is greater in women at rest or combined with exercise; hence, women seem less peripherally limited than men in hypoxia. While sex differences in hematological parameters are well known, they do not impact acute hematological responses to hypoxia (i.e., plasma contraction). Regarding increases in hemoglobin mass, no clear consensus is apparent and the putative sex differences seem due to low iron stores (more common in women). Although these responses are known to be sensitive to sex hormone fluctuations, the effect of menstrual cycle and the influence of menopause on physiological responses to hypoxia remain poorly investigated.

All these responses demand further investigation, with appropriate designs to characterize sex-specific differences. While a growing body of evidence has demonstrated the mechanisms that could impact sex-dependent responses to hypoxia, the impact on performance in hypoxia, mountaineering, and susceptibility to severe altitude illness is not yet fully understood.

Further work is required to translate these sex differences in responses to hypoxia into practical recommendations, either for reducing the risks at high altitude or for improving performance or health benefits associated with altitude training or hypoxic conditioning.
